# A robust electrophysiological marker of spontaneous numerical discrimination

**DOI:** 10.1038/s41598-020-75307-y

**Published:** 2020-10-27

**Authors:** Carrie Georges, Mathieu Guillaume, Christine Schiltz

**Affiliations:** 1 Department of Behavioural and Cognitive Sciences (DBCS), Faculty of Humanities, Education and Social Sciences (FHSE), Institute of Cognitive Science and Assessment (COSA), University of Luxembourg, Campus Belval, Maison des Sciences Humaines, Porte des Sciences 11, 4366 Esch-sur-Alzette, Luxembourg; 2 Center for Research in Cognitive Neuroscience (CRCN), Université Libre de Bruxelles, Avenue Franklin Roosevelt 50 (CP 191), 1050 Brussels, Belgium

**Keywords:** Cognitive neuroscience, Psychology

## Abstract

Humans have a Number Sense that enables them to represent and manipulate numerical quantities. Behavioral data suggest that the acuity of numerical discrimination is predictively associated with math ability—especially in children—but some authors argued that its assessment is problematic. In the present study, we used frequency-tagged electroencephalography to objectively measure spontaneous numerical discrimination during passive viewing of dot or picture arrays in healthy adults. During 1-min sequences, we introduced periodic numerosity changes and we progressively increased the magnitude of such changes every ten seconds. We found significant brain synchronization to the periodic numerosity changes from the 1.2 ratio over medial occipital regions, and amplitude strength increased with the numerical ratio. Brain responses were reliable across both stimulus formats. Interestingly, electrophysiological responses also mirrored performances on a number comparison task and seemed to be linked to math fluency. In sum, we present a neural marker of numerical acuity that is passively evaluated in short sequences, independent of stimulus format and that reflects behavioural performances on explicit number comparison tasks.

## Introduction

We can effortlessly approximate how many people are in a given room or how many objects form a collection. Dehaene postulated that humans have a Number Sense, a cognitive ability that allows representing and manipulating large numerosities^1^. This intuitive understanding of numbers has been defined as a cognitive system dedicated to number processing, the Approximate Number System (ANS^2^). The acuity of this cognitive system is characterized by scalar variability^3^ so that mental representations of large numerosities are less precise than those of small numbers. It is assumed that this system follows the Weber–Fechner law^4–6^, for alternative views. Accordingly, comparing two quantities depends on the numerical ratio between them (e.g., 10 and 20 objects are as distinguishable as 20 and 40 objects). In the literature, ANS acuity is commonly assessed through non-symbolic number comparison tasks, with the underlying idea that better numerical discrimination results from more precise number representations, that is, greater ANS acuity.

Much attention has been devoted to this cognitive system since the observation that ANS acuity predicted adolescents’ arithmetic performance throughout their scholarship^7^. Some studies also reported a close relationship between ANS acuity and mathematical ability (in children, r = 0.54 in^8^, r = 0.35 in^9^, r = 0.52 in adults^10^, but other studies did not report such a correlation (e.g.,^11–14^, for meta-analyses). This discrepancy has been attributed to ambiguities relative to the number comparison task^15–17^. In such a task, participants strategically use all available information from the visual scene to make their decision^18^, see also^19,47^ for extensive discussions about the interplay between numerical and non-numerical processes). This has led some authors to suggest that comparison tasks mostly involve executive processes (notably inhibition) at the expanse of numerical processes^20,21^. The non-negligible involvement of executive functions in number comparison tasks could thus disqualify their validity of indexing basic numerical processes^22^, see also^52^. To adequately measure ANS acuity, one therefore needs the possibility to assess numerical discrimination without any explicit task to thereby prevent deliberate judgements based on non-numerical information.

Recent studies found that humans can spontaneously perceive number from a visual scene, without any explicit task related to number processing^23,24^. These observations support the idea that numerosity is a salient property of the visual environment. It was even further proposed that number is a topologically invariant property, independent from other visual percepts^25^. This view is notably supported by deep-network modeling showing that numerosity emerges as a statistical property of pictures in hierarchical generative models^26^. Burr and Ross already emphasized in 2008 that numerosity is a primary visual property, easily captured by a Visual Number Sense^27^. The ability to extract numerical information from a visual scene is now considered as a universal ability shared with other animal species^28^. Recent neuropsychological data provide evidence that specific visual mechanisms capture numerosity independently of cognitive control^29^. Such visual processes dedicated to numerical extraction could thus be an interesting proxy of ANS acuity and more generally numerical ability.

In the current study, we intended to measure the ability to spontaneously extract numerical information from visual scenes by recording brain responses to numerical fluctuations during a relatively short passive viewing task that did not involve any explicit numerical processes. Furthermore, we aimed at comparing brain responses to such numerical information in the context of either simple geometrical forms (i.e., dot arrays) or richer colourful pictures. To achieve these goals, we adapted a paradigm based on the Fast Periodic Visual Stimulation (FPVS) method^30^.

The FPVS approach is based on the observation that the human brain synchronizes its activity to the periodic state of a flickering stimulus^31^, leading to Steady-State Visual Evoked Potentials^32^. By introducing a periodic fluctuation of a target stimulus feature during a sequence of passively viewed stimuli, we can favourably use this synchronization property of the brain to record cerebral responses specific to the periodic feature manipulation. Indeed, FPVS provides an objective measure of brain sensitivity to the periodic changes of identity^33,34^. Many studies used the FPVS design to investigate neural discrimination of face identities^35,36^, facial expressions^37^, letters and words^38,39^, tool categories^40^, and digits^41^. We recently found^42,43^ that FPVS can also provide a reliable electrophysiological measure of numerical discrimination independent from other visual properties (see also^44,45^,for a similar observation). These findings make FPVS a valuable tool to measure numerical discrimination without requiring any explicit task.

**Figure 1:**
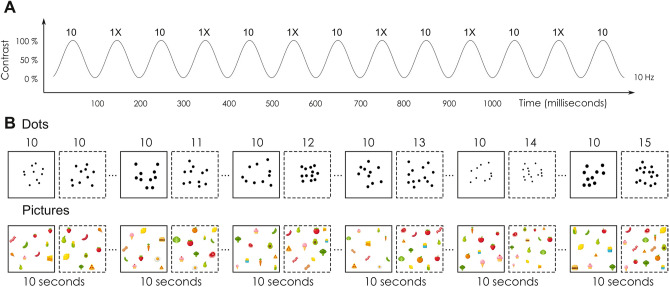
Illustration of the experimental design. (**A**) The onset and the offset of the stimuli followed a sinusoidal contrast stimulation (from blank to full luminance) at 10 Hz. There was a 5 Hz alternation between the carrier (10) and a second number (1×). (**B**) In two different conditions (cautiously designed dots or colourful pictures), the value of the second number increased every ten seconds, from 10 to 15.

In the current study, we recorded electrophysiological responses to periodic changes of numerosity in a fast stream of visual stimuli following a sinusoidal contrast modulation at 10 Hz (see Fig. 1A). We manipulated the number of displayed items so that it systematically switched from ten to another number. The carrier (i.e., ten) alternated with the second number at the fluctuating rate of 5 Hz. The value of the second number progressively increased during the 1-min sequence, from 10 to 15. The numerical increment occurred every 10 s. The numerical ratio between the carrier and the second number thus raised from 1.0 to 1.5 (with a step of 0.1) across six continuous 10-s periods. This method using a progressive numerical increment is a significant improvement of our previous method where we assessed brain responses to numerical ratios in different conditions^42,43^. Here the progressive numerical increment allowed us to determine in one sequence the first ratio at which numerical discrimination was achieved at the brain level. In this respect, electrophysiological responses tagged at 5 Hz (i.e., the frequency of the alternating change) are an individual and objective neural marker of numerical discrimination.

We designed two stimuli sets for the FPVS sequences: a first set of simple but carefully constructed items and a second set intended to be more complex but also more appealing (see Fig. 1B for illustrations). The first set consisted in dark dot arrays with homogeneous dot sizes. For this set, we meticulously controlled, at the sequence level, for unwanted periodic changes related to several potentially interfering visual features (as in^43^. Conversely, the second set was intended to be richer and as such composed of colourful food drawings of heterogeneous sizes. We deliberately did not control for any visual features in this condition. On the contrary, it included even more complex visual changes in that quite a few items contained varying numbers of sub-items (e.g., there are three pepperonis on the pizza picture). Comparing these two sets allowed us to verify whether the neural marker of numerical discrimination is robust to stimulus complexity, thus providing us with a reliable measure of spontaneous numerical information processing.

## Results

### Numerical acuity and math fluency

In the non-symbolic number comparison task, participants were able to determine the larger of the two dot arrays with a mean accuracy of 91.07% (Standard Deviation (*SD*) = 2.93%), and they yielded the correct response on average in 570 ms (*SD* = 77 ms). Accuracies ranged from 77.53 to 98.51% and correct RTs from 647 to 524 ms for the closest (i.e., 1.1) and the most distant (i.e., 1.6) ratios respectively (see Fig. 3C). The mean value of the Weber fraction was 0.116 (*SD* = 0.03). In the math fluency task, average performance across all five subtests was 75% (*SD* = 13.55%), with an averaged raw score of 150 out of 200.

### FPVS recording

#### Instruction compliance

We assessed compliance by instructing participants to keep their gaze on a fixation diamond displayed at the centre of the screen. The diamond randomly (between six to eight times) changed color during the sequences, and participants were instructed to press a button upon detecting the change (as in^42^, see “Methods”). Participants overall detected the color change in 509 ms (*SD* = 50 ms) with only 4.24% (*SD* = 3.41%) of misses across both the dots and pictures conditions. Such a relatively high detection rate (> 95%) indicates that participants kept their gaze on the centre of the screen during the EEG acquisition.

#### Topographies and signal-to-noise ratio

**Figure 2:**
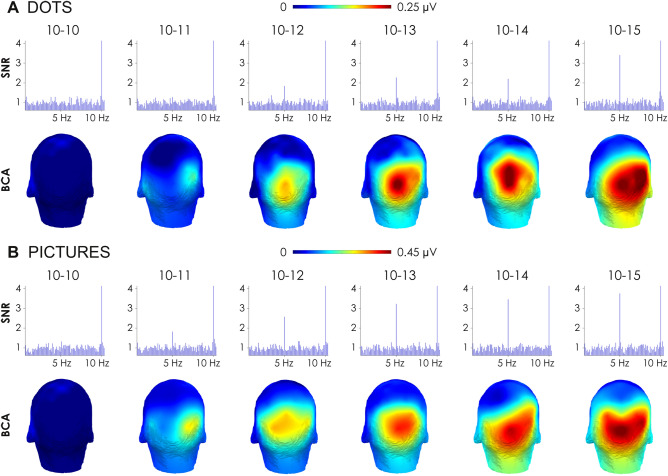
Brain responses to numerical changes for (**A**) the Dots condition and (**B**) the Pictures condition. Each upper part depicts the grand-averaged Signal-to-Noise Ratio (SNR) spectra over the medial occipital electrode Oz for every numerical ratio. Each lower part shows the topographical maps of the 5 Hz Baseline-Corrected Amplitudes (BCA, in μV) for each numerical ratio.

Figure 2 depicts the topographies of the cerebral responses specific to the numerical discrimination of the alternating change. Accordingly, we computed the Baseline-Corrected Amplitudes (BCA, see “Methods”) at the frequency of interest (i.e., 5 Hz) for every ratio (i.e., for each 10-s segment) in each condition (Fig. 2A: Dots condition; Fig. 2B: Pictures condition). We separately computed BCAs for every participant and then averaged the amplitudes at the inter-individual level. No responses at 5 Hz were observed at the scalp level for a ratio of 1 (i.e., no change) in any of the two conditions. Conversely, for numerical ratios equal to and greater than 1.2, strong peaks were recorded in posterior regions, mostly in the medial occipital area centered around the electrodes Iz, O1, O2 and Oz (see Supplementary Table 1 for comparably lower frequency-tagged EEG responses in the left, medial, and right occipito-parietal cortices). Since similar response patterns were observed on each of these medial occipital electrodes (see Supplementary Fig. 1), further analyses were limited to the electrode Oz for reasons of brevity and to be in accordance with previous studies consistently reporting large number-specific effects on that electrode (see e.g.,^42–45^).

Figure 2 also depicts the average Signal-to-Noise Ratio (SNR, see “Methods”) of the EEG spectra recorded on the medial occipital electrode (Oz) for every ratio in the dots and pictures conditions. SNR peaked at 10 Hz for every ratio in each condition (across all ratios: dots: mean = 7.46, *SD* = 0.38; pictures: mean = 7.68, *SD* = 0.32). This is expected since these cerebral responses reflect the visual contrast between the background and the stimuli induced by their periodic onset at 10 Hz. Most importantly, there was a very clear brain response at 5 Hz on Oz associated with a numerical ratio equal to and greater than 1.2 for both dots and pictures. The SNR seemed to progressively increase with increasing numerical ratio in both conditions.

#### Quantification of the response

To assess the statistical significance of the cerebral responses synchronised to numerical changes, we computed a Z-score of the response at 5 Hz for every ratio in each condition per participant (see “Methods”). A Z-score value larger than the threshold of 1.64 (*p* < 0.05, one-tailed, testing signal level > noise level) indicates a significant cerebral response to the number change, and thus measures successful numerical discrimination at the neuronal level.

**Figure 3:**
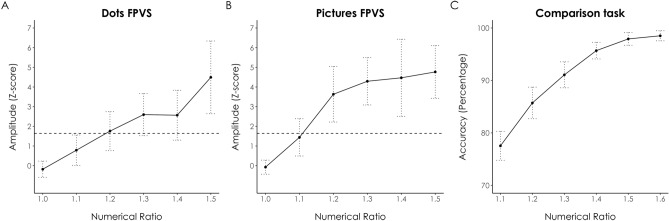
EEG and behavioural data depiction. Amplitudes of Oz (in Z-score) in (**A**) the Dots condition and (**B**) the Pictures condition as a function of the numerical ratio. The horizontal line depicts the 95% significant threshold (i.e., 1.64) above the noise level. (**C**) Behavioural data from the non-symbolic number comparison task (accuracy, in percent). Vertical lines depict 95% Confidence Intervals in each graph.

We found that the EEG signal tagged at 5 Hz on the medial occipital electrode Oz averaged across all participants was significantly above the noise level from a numerical ratio of 1.2 onwards in both the dots and pictures conditions (see Fig. 3A,B). This suggests that visual number discrimination was successfully achieved from the ratio 1.2. Note that this finding is consistent with our behavioural data yielding an averaged Weber fraction value of 0.116. This value indicates accurate (i.e., more than 75% correct) number discrimination from a ratio of 1.116 onwards, corresponding to significant EEG responses at the 1.2 ratio, which was the next ratio sampled after 1.1 in our EEG design. In other words, our FPVS results are comparable to the behavioural performances on the number comparison task, indicating accuracies significantly greater than 75% from a ratio of 1.2 onwards (see Fig. 3C). Further, EEG signal amplitudes seemed to progressively increase with increasing numerical ratio in both conditions.

Overall, the tagged cerebral response on the medial occipital electrode Oz was stronger for pictures (Z-score = 3.09, *SD* = 3.32) than dots (Z-score = 2.00, *SD* = 2.89). We conducted linear mixed effect model analyses to determine whether the brain responses depended on the condition and/or the numerical ratio. We entered the condition and the ratio as fixed effects, with an interaction term, and we included intercepts for participants as random effect. Model comparisons between the full model and reduced models were done using chi-squares tests on the log-likelihood values. Since the full model did not provide a better fit than the reduced model without the interaction term, χ^2^(1) = 0.60, p = 0.44, we proceeded with contrasting the model without interaction term to models that were further reduced by excluding the remaining effects in question. Model fits were significantly worse when excluding either condition, χ^2^(1) = 14.82, p < 0.001, or ratio, χ^2^(1) = 99.91, p < 0.001, which indicates that both factors (i.e., condition and the numerical ratio) had significant effects on the brain response.

To further assess the effect of numerical ratio on the tagged cerebral responses, and to verify whether the apparent increase in amplitudes across the ratios was significant, we conducted linear regression analyses to predict the EEG signal at 5 Hz on Oz as a function of numerical ratio. Results indicated significant positive trends for both dots (adjusted r^2^ = 0.93, β = 8.44, p < 0.001) and pictures (adjusted r^2^ = 0.82, β = 9.7, p = 0.008). The cerebral response reflecting numerical discrimination thus progressively increased with increasing numerical ratio in both conditions (see Fig. 3A,B).

#### Reliability of the response

To confirm the robustness of the aforementioned group findings at the individual level and thereby the sensitivity of the current approach, we assessed both inter- and intra-individual reliabilities of the EEG responses at 5 Hz.

**Figure 4:**
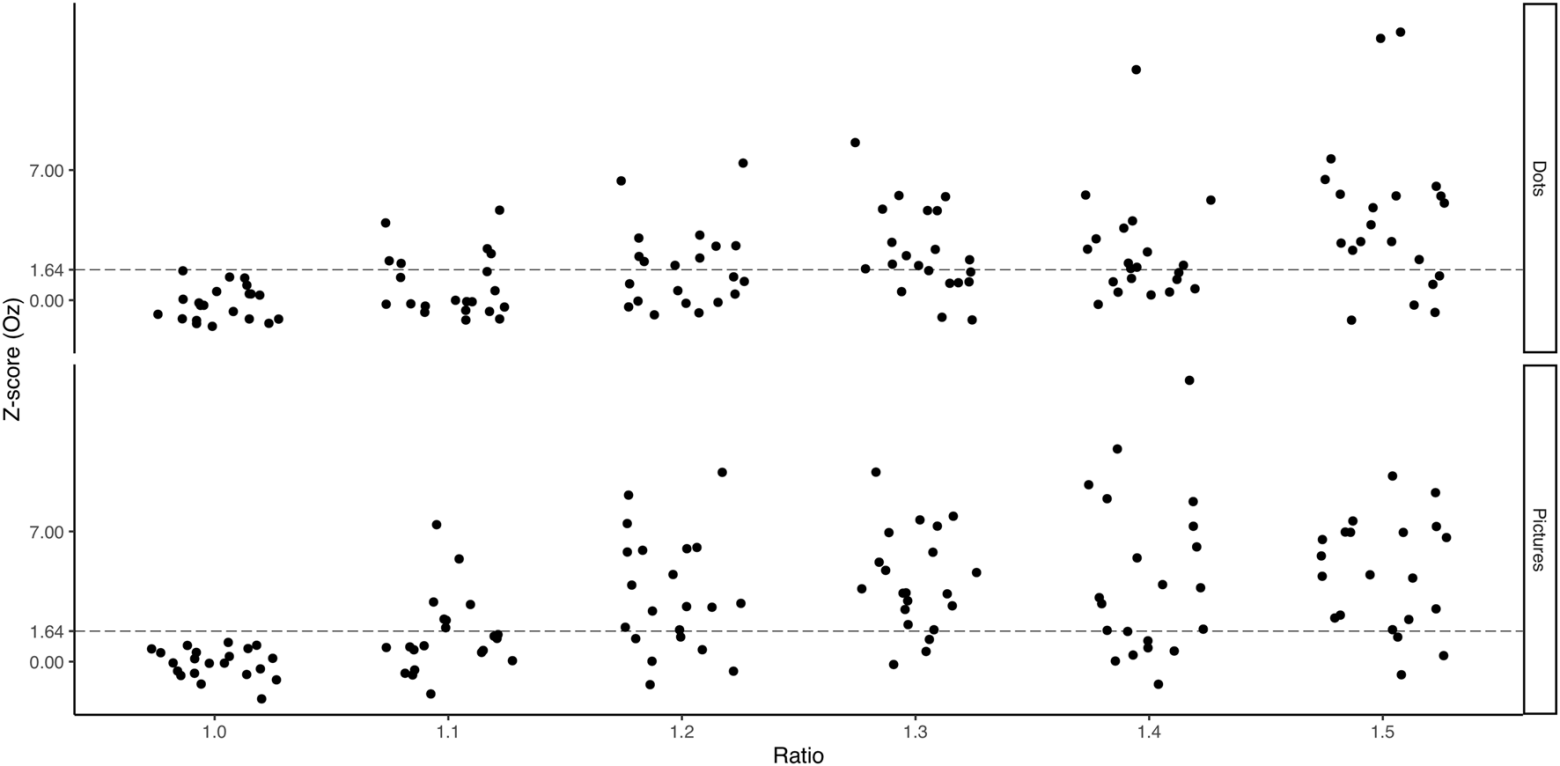
Individual amplitudes of Oz (in Z-score) as a function of the condition (Dots or Pictures) and the numerical ratio. The vertical dashed line represents the 1.64 threshold of statistical significance at 95%.

In terms of inter-individual reliabilities, 48% and 71% of the participants featured a significant frequency-tagged EEG signal (i.e., Z-score > 1.64) on Oz at the numerical ratio 1.2 (i.e., the smallest ratio at which on average significant cerebral responses to numerical discrimination were recorded on Oz at the group level) in the dots and pictures condition, respectively. This agrees with Guillaume et al.^42^, reporting number-tagged effects in brain amplitudes also in the majority of their participants and clearly suggests that the present group-level cerebral response at 5 Hz on Oz at the numerical ratio 1.2 was not driven by a few outlier participants. The proportion of participants featuring significant frequency-tagged EEG signals on Oz also gradually increased with increasing numerical ratio (see Fig. 4). This is to be expected if one assumes that numerical discrimination, as reflected by significant EEG responses at 5 Hz on Oz, becomes progressively easier with increasing numerical ratio.

It should be noted that when considering for each individual the medial occipital electrode (i.e., Iz, O1, O2, or Oz) at which the largest frequency-tagged response was observed at the ratio 1.2 (instead of focussing on Oz in every individual), significant numerical discrimination was observed in 57% and 90% of the participants at that numerical ratio in the dots and pictures conditions, respectively. When considering the entire posterior scalp, significant cerebral responses at 5 Hz for the numerical ratio 1.2 were observed in 67% and 100% of participants with dots and pictures stimuli, respectively. These data generally confirm the robustness of the present group-level findings at the inter-individual level.

In terms of intra-individual reliabilities, we also focussed on frequency-tagged cerebral responses on Oz at the numerical ratio 1.2, considering that it was the lowest ratio yielding significant frequency-tagged EEG signals at the group-level. Intra-individual reliabilities were computed using the odd–even split-half method based on 10 repetitions per condition (see also “Methods”). More concretely, two EEG amplitudes (in Z-score) were computed at 5 Hz for every individual in each condition and subjected to correlation analysis. The correlation coefficients were Spearman–Brown corrected to get a reliability estimate for the entire set of items (i.e., 10 repetitions). Spearman–Brown corrected correlation coefficients were r = 0.33, p = 0.41 and r = 0.68, p = 0.02 in the dots and pictures condition, respectively. When excluding one influential data point with a Cook’s distance greater than the conventional cut-off value of 1.0 (i.e., with Cook’s distance = 2.1)^46^, intra-individual reliability in the dots condition improved to r = 0.66, p = 0.03. This thus suggests that within-subject reliabilities of the EEG responses reflecting numerical discrimination were within the acceptable range in both the dots and pictures conditions.

### Relationship between the FPVS response and the behavioural measures

**Figure 5:**
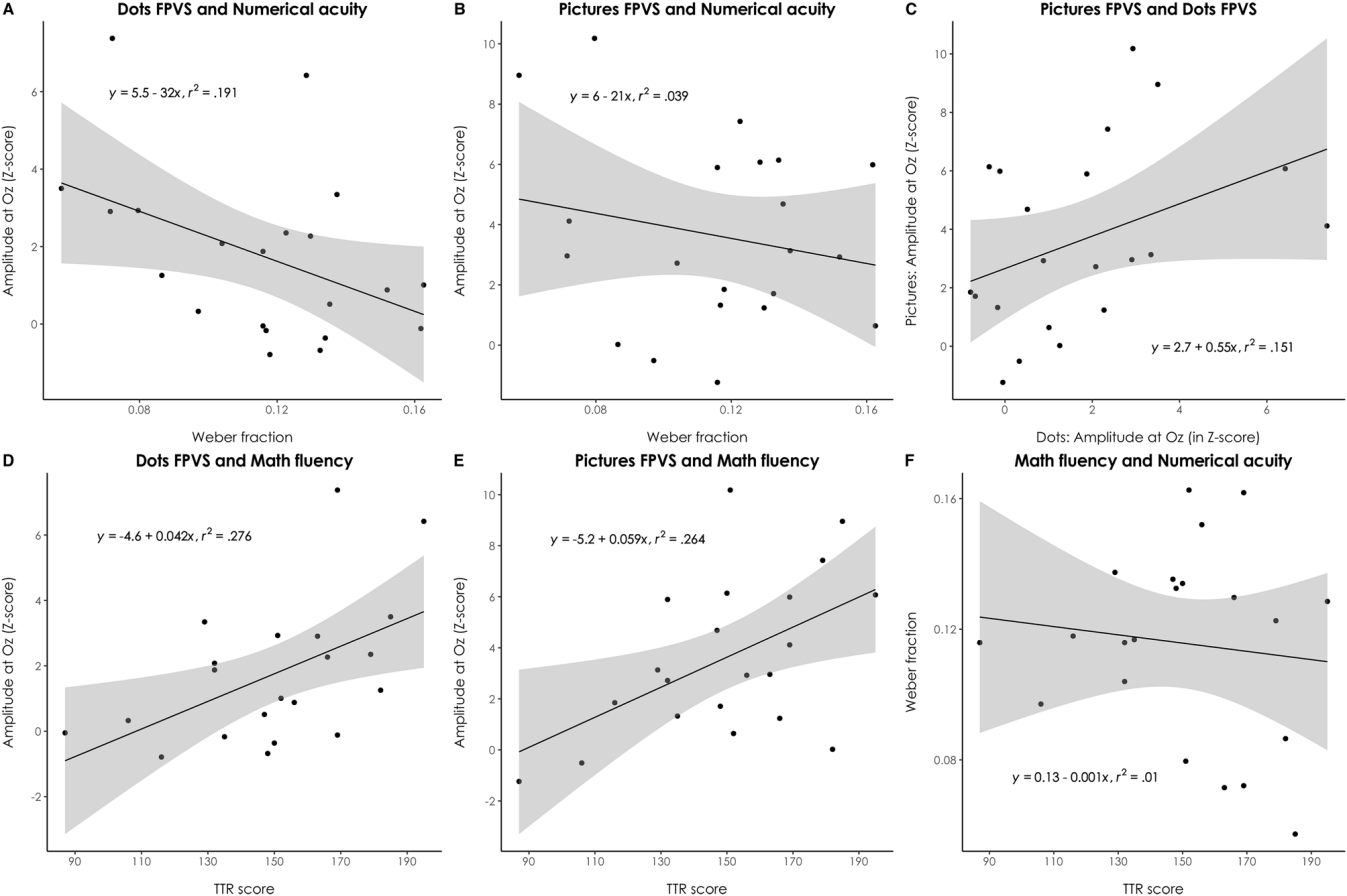
Correlations between EEG and behavioural data. Scatter plots of (**A**) the values of the Weber fraction and the amplitudes recorded on Oz for the numerical ratio 1.2 in the Dots condition and (**B**) the Pictures condition, (**C**) the amplitudes recorded on Oz for the numerical ratio 1.2 in the Dots and Pictures conditions, (**D**) the math fluency score and the amplitudes recorded on Oz for the numerical ratio 1.2 in the Dots condition and (**E**) the Pictures condition, (**F**) and the values of the Weber fraction and the math fluency scores. The equation of the linear regression line is indicated in each plot. Gray areas depict standard errors.

Although this was not one of the main objectives of the present study, we conducted correlation analyses to test whether the EEG signal reflecting numerical discrimination was related to a behavioural measure of numerical discrimination as well as math fluency. Stronger cerebral responses at 5 Hz on the medial occipital electrode Oz at the ratio 1.2 in both the dots and pictures conditions were associated with significantly better math fluency (dots: *r* = 0.53, *p* = 0.014; pictures: *r* = 0.51, *p* = 0.017; see Fig. 5D,E). Conversely, only in the dots condition, a relation was observed between higher EEG signals at 5 Hz on Oz at the ratio 1.2 and lower Weber fractions (dots: *r* = − 0.44, *p* = 0.047; pictures: *r* = − 0.20, *p* = 0.39, see Fig. 5A,B). Notably, the brain responses in the dots condition only marginally correlated with those in the pictures condition (*r* = 0.39, *p* = 0.08; see Fig. 5C), suggesting common but also distinct brain processes related to the stimulus complexity. It should also be noted that the values of the Weber fraction did not correlate with math fluency scores in the present study (*r* = − 0.12, *p* = 0.62; see Fig. 5F). When correcting the aforementioned correlations for multiple comparisons using the Holm-Bonferroni step-down procedure, only relations between stronger EEG signals at 5 Hz on Oz at the ratio 1.2 in both the dots and pictures conditions and better math fluency remained significant, when considered one-tailed (dots: *p* = 0.042; pictures: *p* = 0.043).

Further correlation analyses using different neural indices of numerical discrimination can be found in the supplementary material.

However, considering the small sample size of the current study for correlation analyses, we acknowledge that the present brain-behaviour relations should be considered with caution without drawing strong conclusions from their outcomes. We therefore complemented the correlation analyses with group comparisons. Since one of the main objectives of the current study was to determine the earliest ratio for which numerical discrimination was achieved at the brain level using both dots and pictures stimuli, we proceeded by distinguishing individuals based on whether they already featured significant cerebral responses (i.e., Z-score > 1.64) at 5 Hz on Oz at the early ratio 1.2. We conveniently chose this ratio because 48% of the participants showed significant discrimination (henceforth referred to as “significant signal at 1.2”), while 52% did not (henceforth referred to as “non-significant signal at 1.2”) in the dots condition. We used this balanced grouping index as between-subject factor in an independent t-test analysis. Individuals with significant EEG responses at 1.2 featured significantly better performances in the number comparison task both in terms of overall accuracy (significant signal at 1.2: 92.5% vs. non-significant signal at 1.2: 89.77%; t(19) = − 2.36, p = 0.015, Cohen’s d = − 1.08, one-tailed) and of the Weber fraction (significant signal at 1.2: w = 0.102 vs. non-significant signal at 1.2: w = 0.128; t(19) = 2.26, p = 0.018, Cohen’s d = 1.04, one-tailed). This group of individuals also showed significantly better math fluency (significant signal at 1.2: sum of correct responses = 160 vs. non-significant signal at 1.2: sum of correct responses = 141; t(19) = − 1.71, p = 0.05, Cohen’s d = − 0.78, one-tailed). Here, it might also be worth noting that when performing a median-split on math fluency scores, only individuals with higher math fluency (i.e., scores above the median value of 151) showed on average significant EEG responses at 5 Hz on Oz at the numerical ratio 1.2 (Z-score = 2.79 compared to 0.61 in individuals with math fluency scores below the median value of 151). These findings thus collectively replicate the aforementioned correlation outcomes.

We were not able to perform such group comparisons for the pictures condition because here 71% of the participants showed a significant EEG signal at 5 Hz on Oz at 1.2, and similar grouping would be unbalanced. In any case, in the pictures condition, cerebral responses reflecting numerical discrimination at 1.2 were significantly stronger in those individuals that already showed a significant EEG signal at 5 Hz on Oz at the ratio 1.2 in the dots condition (significant signal at 1.2: 5.27 μV vs. insignificant signal at 1.2: 2.14 μV; t(19) = − 2.63, p = 0.009, Cohen’s d = − 1.21, one-tailed).

## Discussion

In the present study, we were able to extract a cerebral measure of numerical discrimination using an implicit task that only involved passive viewing of numerical quantities, without requiring any explicit numerical judgements. At the group level, participants significantly discriminated between non-symbolic numerosities from the ratio of 1.2 onward. Interestingly, this ratio corresponded to the averaged behavioural performance on the number comparison task, where accuracies were significantly greater than 75% correct from 1.2 onward. The current study thus successfully yielded an objective neural marker of ANS acuity that directly reflects explicit numerical discrimination in behavioural tasks. Since our marker was extracted during very short sequences, the present study also significantly extents previous observations (e.g.,^42^) by considerably reducing the amount of time required to determine the sensitivity to numerical discrimination.

Topographical maps revealed that the strongest responses to the periodic numerical changes (at 5 Hz) were located in medial occipital regions, with response patterns being very similar between the medial occipital electrodes Iz, O1, O2, and Oz. This is in accordance with previous studies, indicating that numerical information is already extracted at lower levels by visual processes^23,24,27,43,44^.

Crucially, the magnitudes of the medial occipital responses linearly increased with the magnitudes of the deviant numerosity (nicely reflecting the architecture of the ANS^4^). Since we progressively increased the deviant magnitudes during the FPVS sequence, we were able to track the evolution of the brain responses to the periodic number changes at the individual level. We then used the amplitudes of the responses recorded on Oz to get an objective neural marker of spontaneous numerical discrimination. Such a marker is desirable because traditional comparison tasks are influenced by non-numerical aspects and involve unwanted deliberate executive processing (e.g.,^18,19,21^). At the group level, we found that the first ratio where numerical discrimination was achieved at the brain level was 1.2 in the present sample. At that ratio, almost half of the participants (48%) showed significant numerical discrimination in the dots condition, and a little less than three quarters (71%) significantly discriminated numerical quantities in the pictures condition, with EEG response amplitudes being relatively reliable at the intra-individual level regardless of stimulus format. It should be noted here that previous works highlighted comparable responses only from a ratio of 1.4 onwards^42,43^. This slight discrepancy might be explained by the present participants’ high ability to discriminate between numerosities, which was reflected by the comparably low Weber fraction of 0.116 in the number comparison task (this value is on average 0.22 in typical adult samples^62^. Such an elevated sensitivity to numerical discrimination at the behavioural level in the present sample might then account for the observation that the cerebral responses capturing the numerical change already reached significance at the group level at the early ratio of 1.2.

Interestingly, individuals featuring significant brain synchronisation at a ratio as low as 1.2 in the dots condition performed better on the number comparison task (as reflected by lower Weber fractions) than individuals whose brain did not discriminate this ratio. This observation was confirmed by correlations analyses (albeit bearing in mind the relatively small sample size of the current study for such analyses), in that participants with a greater neural sensitivity to numerical changes using controlled dots featured better behavioral ANS acuity (as in^42^). This finding supports that cognitive processes indexing spontaneous numerical discrimination recorded with EEG are related to explicit numerical judgements. It should, however, be noted that no significant correlation was observed between ANS acuity and the neural sensitivity to numerical discrimination when using pictures (instead of controlled dots) as stimuli. Apart from null findings related to small sample size, an explanation for this discrepancy could be differences in the stimuli formats. Using the same stimuli in the number comparison task than in the controlled dots condition might have favoured the relation between brain sensitivity and behavioural performance. If this explanation applies, using more complex and colorful stimuli instead of dots in the behavioural task should then favour a correlation with the neural sensitivities to numerical changes in the pictures condition. In any case, the current correlation outcomes should be considered with caution and verified using larger sample sizes.

It is worth mentioning that brain amplitudes tagged to the numerical fluctuation were systematically stronger in the pictures condition than in the dots condition in our dataset. In other words, pictures elicited stronger synchronisation to the numerosity changes. This is not totally surprizing because we deliberately did not control for potential confounding factors related to non-numerical dimensions in this condition^19,47^. It is likely that visual information inherently correlated with numerosity provided additional congruent information regarding the numerical aspect of the arrays. On the contrary, it does not seem that the complexity of the pictures (such as containing sub-element) impeded brain synchronization to numerosity. This is in line with existing data showing that number is a salient property of the visual environment^23–25^.

Nonetheless, despite the aforementioned differences between controlled dots and colorful pictures, similar electrophysiological response patterns were observed regardless of stimulus complexity, suggesting that both conditions indexed ANS acuity and can therefore be used as neural signatures of the number sense. More concretely, we observed a significant effect of ratio in both conditions. In addition, cerebral responses reflecting numerical discrimination were on average significant from the ratio of 1.2 onward for both dots and pictures stimuli, which also corresponds to the ratio at which averaged behavioural performances were significantly above 75% correct in the number comparison task. Moreover, the neural sensitivities to numerical changes were related across both conditions. Namely, cerebral responses in the dots condition tended to correlate with those in the pictures condition, indicating common brain processes regardless of stimulus format. Overall, these findings suggest that the neural marker of numerical discrimination is quite robust to stimulus format in an inter-individual perspective. This is a promising avenue for future research, as appealing pictures instead of controlled dots could be used to measure ANS acuity. The possibility to use appealing stimuli together with the fact that the neural marker is extracted using a passive viewing task of very short duration makes the present paradigm especially suitable for younger populations including infants, which are usually easily distracted.

A final interesting finding is that the neural sensitivities to numerical discrimination related to a measure of math fluency regardless of whether dots or appealing pictures were displayed. Since the neural marker of ANS acuity was extracted using a passive viewing task devoid of any explicit numerical judgments, these findings further hint at the importance of the number sense for mathematical development. Nonetheless, it should be noted here that although the correlations between the frequency-tagged EEG signal and math fluency were corrected for multiple comparisons as well as confirmed with group comparisons, they should be considered with caution due to the relatively small sample size of the present study and replicated with more participants to validate the present outcomes. In spite of a power analysis justifying the current sample size for the extraction of a neural marker of numerical discrimination using very short sequences as well as for the assessment of any potential effects of stimulus format (i.e., dots versus pictures), it was likely too small to draw any strong conclusions from the correlation analyses.

Our small sample size of n = 21 could then also account for the lack of correlation between the behavioural performances on the non-symbolic number comparison task and math fluency, since the effect size for this relation was reported to be below 0.3 (see^13,14^, for meta-analyses). Albeit, such a null correlation would also be in line with many previous studies that failed to report a significant relation between ANS acuity, as assessed using non-symbolic number comparison tasks, and math ability in adults (e.g.,^8,13,14^). Considering that more general cognitive processes (e.g., executive control functions, see^20,21^) were suggested to potentially moderate the relation between explicit measures of the number sense and math fluency, further studies should investigate the potential role of executive functions in the relationship between spontaneous visual number processing and more elaborate mathematical knowledge. It goes without saying that these studies should focus on larger sample sizes as well as different populations, including children. Finding any significant relations between the cerebral responses reflecting numerical discrimination and explicit numerical processing as well as math fluency in the latter individuals would open up the practicality of the present paradigm even further, by suggesting that it could be used to predict math ability in young children. In this regard, it should also be verified whether frequency-tagged EEG signals reflecting numerical discrimination can predict math fluency in out-of-sample participants, as suggested by Gabrieli et al.^48^.

In conclusion, we present a rapid and reliable method providing a neural marker of ANS acuity that is independent of stimulus format and reflects behavioural performances on explicit number comparison tasks.

## Methods

### Participants

Twenty-two students from the University of Luxembourg participated in this study. Volunteers suffering from or with a history of suffering from any neurological or neuropsychological disease, any learning disability such as dyscalculia, or any uncorrected visual impairment were not allowed to participate. All participants gave written informed consent and received a remuneration of 25 euros. We excluded one participant from all analyses who did not adequately respond to the behavioural task during the EEG recording. The final sample thus consisted of twenty-one adults (eight males), with a mean age of 23.43 years (*SD* = 4.46, range 18.23–35.40).

A statistical power analysis was conducted using the G*Power 3 software^49^ to estimate the current sample size. The power analysis was conducted for a one-way repeated measures ANOVA including two levels, since we were interested in whether significant frequency-tagged EEG signals on Oz differed depending on stimulus format (i.e., dots versus pictures). The correlation between the repeated measures was set to 0.5, the default value of G*Power, since we did not have any a priori hypotheses regarding the correlation between EEG signals recorded during the presentation of dots or pictures. Estimation was based on previously reported effect sizes in an EEG study aimed at determining a neural index of numerical sensitivity in adults (see^44^). In that study, large number-specific effects (i.e., ƞ_p_^2^ = 0.186) were observed amongst others on the medial occipital electrode Oz. With an alpha = 0.05 and power = 0.95, a sample size of n = 17 is sufficient to detect effects at this level for comparisons between two repeated conditions. The current sample size of n = 21 is therefore more than adequate for the main objectives of this study, that is, (1) the extraction of a cerebral measure of numerical discrimination from posterior brain regions using very brief sequences and (2) the assessment of the dependence of this neural marker on stimulus format (i.e., dots versus pictures).

### Apparatus and procedure

We used MATLAB (The MathWorks) with the Psychophysics Toolbox extensions^50,51^ to display the computerized tasks and record behavioural data. Participants were comfortably seated at 1 m from the screen, with their gaze directed at the centre of the screen (24” LED monitor, 100 Hz (Hz) refresh rate, 1 ms response time). Screen resolution was 1280 × 1024 px. Behavioural tasks were administered first. All participants started with the math fluency task. After that, they took part in the EEG recording session, consisting of two non-symbolic number conditions administered in counter-balanced order across the participants.

### Non-symbolic number comparison task

Participants simultaneously saw two dot arrays and they were instructed to determine as accurately as possible the array containing the largest number of dots. The onset of each trial was indicated by a fixation cross appearing 500 ms before the dots. The arrays only remained on the screen for a maximal duration of 800 ms to prevent participants from counting the dots. We used an active mask until response to suppress retinal persistence. Inter-stimulus interval lasted 400 ms.

We generated non-symbolic number pairs with the help of NASCO^52^. Stimulus pairs were divided into four categories. In a first category, total Area and convex Hull were equalized across the stimulus pair; in a second category, total area and mean occupancy were equalized; in a third category, item size and convex hull were equalized; and in the final category, item size and mean occupancy were equalized. In total, 192 stimulus pairs were generated, each comprising an array of 30 dots as the standard quantity to which the second array (of the pair) had to be compared. The corresponding arrays were created by computing six numerical ratios (from 1.1 to 1.6 with an incremental step of 0.1), starting from the standard quantity of 30 dots in both increasing and decreasing manners. The number of dots in the second array thus ranged from 19 to 48. There were thirty-two pairs for each of the six ratios (i.e., sixteen where the second quantity was below and above 30 respectively). All dots had the same size within an array. Stimulus order and the position of the correct response (either left or right) were randomized.

We computed individual Weber fractions (*w*) by adjusting a Gaussian cumulative probability distribution function using nonlinear regression, based on the Levenberg–Marquardt–Fletcher nonlinear least square iterative method (as in^7,42,53^, for more detailed methodological considerations). We considered *w* as a behavioural measure of explicit numerical judgments.

### Math fluency task

We used the Tempo-Test Rekenen (*TTR*^54^) to evaluate math fluency. This timed paper-and-pencil calculation test consists of five columns of forty arithmetic problems, with each column corresponding to one arithmetic operation (addition, subtraction, multiplication, division) and to a mix of all operations. The item difficulty increases throughout each column, from single-digit arithmetic facts to more complex two-digit problems. Each column was presented on a separate sheet of paper and participants were instructed to write down as many correct responses as they could within one minute. Participants were awarded one point per correct answer. We considered the raw sum of correct responses across all five subtests (max = 200) as a measure of math fluency.

### Fast periodic visual stimulation

#### Material and procedure

Participants were instructed to keep their gaze on a small fixation diamond that was continuously displayed at the centre of the screen. We used stimuli that subtended a maximal visual angle of nine degree. Stimulus presentation followed a sinusoidal contrast modulation from 0 to 100% (see Fig. 1A, as in^40^, or in^55^). The base frequency rate was 10 Hz, corresponding to the display of 10 stimuli per second. Every stimulation sequence lasted 64 s, including 60 s of recording and 2 s of fade-in and fade-out respectively, which were not analysed.

During each stimulation sequence, we alternated between two numerical quantities. Alternation thus occurred at a frequency rate of 5 Hz. One of the numerical quantities used during alternation (i.e., the carrier) was kept constant at a numerosity of 10 items. The second numerosity changed every ten seconds, thus in total 6 times during each stimulation sequence of 60 s. During the first 10 s, the interspersed numerical quantity was identical to the fixed numerosity. It then linearly increased from a ratio of 1.1 (i.e., eleven items) to a ratio of 1.5 (i.e., fifteen items) with an incremental step of 0.1. This increasing fluctuation within a single stimulation sequence is based on the sweep visual evoked potential technique^32,56–59^.

We created two conditions. In the first condition, we presented randomly arranged dots, created with NASCO^52^. We statistically verified that random fluctuations related to the non-numerical visual dimensions were not periodic within our stimulation sequences of 60 s by computing the Fast-Fourier Transformation (FFT) of the values taken by all dimensions over the time (see the supplementary material from^43^). For each condition, we selected one stimuli sequence in which the averaged periodicity value of the numerical dimension was significant whereas the averaged periodicity values of all other non-numerical dimensions were non-significant. In this setting, visual features were thus drastically controlled.

In the second condition, we did not display controlled stimuli, but instead used pictures bought on the Fotolia database. These pictures consisted of colourful food items, such as fruits and vegetables (see Fig. 1). Pictures were randomly drawn (without replacement) to form an array with the desired number of food items. To increase variability, we let the size of each item stochastically vary (25% of variation from its base size, which was heterogeneous as a function of the item) and we also added some random mirroring effect. Importantly, we deliberately choose food items that might contain sub-elements such as green beans (containing three pods) or pizza slices (containing three pepperonis). Therefore, and conversely to our first condition, this second condition was not designed to control for visual cues but rather illustrate stimulus complexity.

Both conditions were repeated ten times in every participant, entailing twenty stimulation sequences (i.e., 20 min of recording) per participants.

To verify that participants kept their gaze at the centre the screen, the small fixation diamond randomly changed colour from blue to red. Colour changes occurred from six to eight times during a stimulation sequence. Participants were instructed to press a button with their right forefinger. Based on these presses, we were able to verify participants’ compliance with the task instruction.

#### EEG acquisition

The setup was similar to the one used in Guillaume et al.^42^. We acquired EEG data using a 64-channel BioSemi ActiveTwo system at 2048 Hz (BioSemi B. V., Amsterdam, The Netherlands). We positioned the electrodes on the cap according to the standard 10–20 system locations (for position coordinates, see https://www.biosemi.com). We used two additional electrodes, the Common Mode Sense (CMS) active electrode and the Driven Right Leg (DRL) passive electrode, as reference and ground electrodes, respectively. We hold offsets of the electrodes below 40 mV during acquisition.

#### EEG analysis

Analyses were conducted with *Letswave 6* (https://nocions.webnode.com/letswave). Data files were down-sampled from 2048 to 512 Hz for faster processing. We then filtered the data with a 4-order band-pass Butterworth filter (0.1 to 100 Hz). In three participants, one channel had to be interpolated across all conditions using the three closest neighbouring electrodes due to excessive noise in the signal throughout the entire EEG recording. We did not correct the EEG signal for the presence of ocular artefacts. The data was then re-referenced to the common average.

We segmented the signal into six chunks of 10 s, corresponding to the six different numerical quantities displayed in alternation with the fixed numerosity of 10 items. The EEG signal from each of the six different ratios with respect to the fixed quantity was then averaged across the ten repetitions for each of the two conditions per participant. A Fast Fourier Transform (FFT) was applied to the signal to extract amplitude spectra for the 64 channels with a frequency resolution of 0.1 Hz.

Based on these frequency spectra, we computed three measures to determine whether and how the brain specifically responded to the alternation of numerical quantities at 5 Hz during each of the two conditions. First, we computed Baseline-Corrected Amplitudes (BCA) by subtracting from the 5 Hz bin the mean amplitude of its twenty surrounding bins (ten on each side, excluding the immediately adjacent bins, and the two extreme values). BCA are thus expressed in microvolt and can therefore be considered to quantify changes within the EEG signal^35,36,60–62^. BCA were used to depict the scalp topographies in Fig. 2.

As a second measure, we computed Signal-to-Noise Ratio (SNR) by dividing each frequency bin with the average amplitude of its respective twenty surrounding bins (excluding the immediately adjacent bins, and the two extreme values). We used SNRs to illustrate the spectra in Fig. 2.

Lastly, we computed a Z-score to quantify the statistical significance of the brain response to the numerosity change at 5 Hz. More concretely, we applied a Z-transformation to the 5 Hz bin as a function of its surrounding twenty bins representing the noise level. This computation yielded a Z-score of the brain response specific to the experimental manipulation at 5 Hz, which can be interpreted as the neural response to the quantity change (i.e., numerical discrimination). A Z-score larger than the threshold of 1.64 (*p* < 0.05, one-tailed, testing signal level > noise level) indicates a significant response to the experimental manipulation. The Z-scores were used to conduct all statistical analyses.

### Ethical statement

We followed APA ethical standards to conduct the present study. The Ethic Review Panel from the University of Luxembourg (ERP) approved the methodology and the implementation of the experiment before the start of data collection.

## Supplementary information

Supplementary Video 1.

Supplementary Video 2.

Supplementary Information 1.

## Data Availability

All data will be made available upon request.
