# Supplementary material for: A robust electrophysiological marker of spontaneous numerical discrimination
Source: Sci Rep. 2020 Oct 27;10:18376. doi: 10.1038/s41598-020-75307-y (PMC7591903; doi:10.1038/s41598-020-75307-y)
Supplement: Supplementary file 3 — Supplementary Information 1. [file 41598_2020_75307_MOESM3_ESM.docx]

**A robust electrophysiological marker of spontaneous numerical discrimination**

Carrie Georges^a*^, Mathieu Guillaume^b^, & Christine Schiltz^a^

**Supplementary Material**

**FPVS recording**

**Instruction compliance for each condition**

Repeated measures ANOVAs indicated a main effect of condition (dots vs. pictures) on both detection rate (F(1, 20) = 21.99, p < .001, η_p_^2^ = .52) and RT (F(1, 20) = 49.91, p < .001, η_p_^2^ = .71). Participants missed fewer color changes in the dots condition (dots: misses = 0.62%, SD = 1.0% vs. pictures: misses = 7.85%, SD = 6.87%) and they were also faster to detect these changes when dots were displayed (dots: RT = 467 ms, SD = 44 ms vs. pictures: RT = 552 ms, SD = 67 ms).

**Response on medial occipital and other posterior electrodes**

To compare frequency-tagged EEG responses in the medial occipital cortex to the remaining posterior areas, we pooled the EEG signal from all the posterior electrodes into four regions of interest (ROIs): the medial occipital (MO: O1, O2, Oz, Iz), medial occipito-parietal (MOP: Pz, POz, PO3, PO4), left occipito-parietal (LOP: P5, P7, P9, PO7) and right occipito-parietal (ROP: P6, P8, P10, PO8) regions (Liu-Shuang, Norcia, & Rossion, 2014). Supplementary Table 1 depicts the Z-score values averaged across all participants for each ratio and condition in these four posterior ROIs. The strongest cerebral responses at 5 Hz across all numerical ratios were on average observed over medial occipital electrodes in both the dots and pictures conditions. Similar frequency-tagged cerebral response patterns, in terms of Baseline-Corrected Amplitudes (BCA, see Methods), across the different ratios were observed on each of the medial occipital electrodes (i.e., Iz, O1, O2, and Oz) in both the dots and pictures conditions (see Supplementary Figure 1).

**Supplementary Table 1.** Averaged cerebral amplitudes (in Z-score) for every ratio in each condition as a function of the posterior region of interest including the medial occipital (MO: O1, O2, Oz, Iz), medial occipito-parietal (MOP: Pz, POz, PO3, PO4), left occipito-parietal (LOP: P5, P7, P9, PO7) and right occipito-parietal (ROP: P6, P8, P10, PO8) regions. Averaged cerebral amplitudes (in Z-score) are also depicted for each of the electrodes comprised in the MO region, yielding the strongest responses in the posterior scalp.


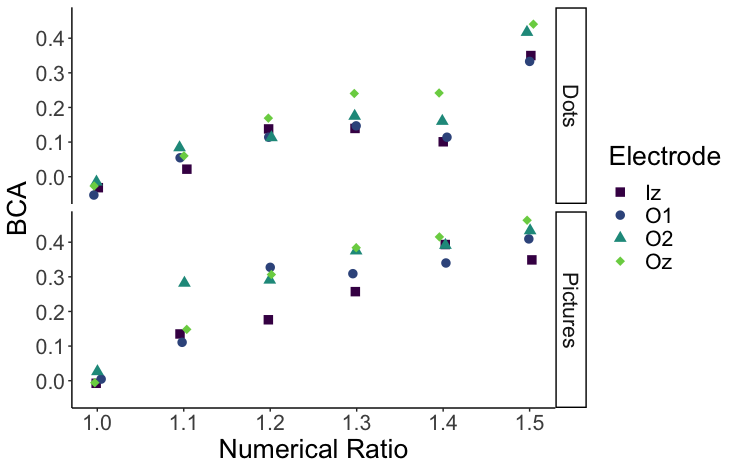


**Supplementary Figure 1.** Averaged cerebral amplitudes (in terms of Baseline-Corrected-Amplitudes, BCA, in μV) on the medial occipital electrodes Iz, O1, O2, and Oz as a function of numerical ratio in the Dots and Pictures conditions.

**Relationship between the FPVS response and the behavioural measures**

To further assess the relations between the EEG signal reflecting numerical discrimination and the behavioural measures, we additionally performed correlation analyses with frequency-tagged cerebral responses at the numerical ratio 1.2 on each individual’s best responding medial occipital electrode at that ratio. All correlations remained similar to when Oz was considered across the entire sample. Namely, in both the dots and pictures conditions, stronger cerebral responses at 5 Hz on each individual’s best responding medial occipital electrode at 1.2 were associated with better math fluency (dots: *r* = .41, *p* = .06; pictures: *r* = .46, *p* = .04). Conversely, only in the dots condition, a relation was observed between higher EEG signals at 5 Hz on every individual’s best responding medial occipital electrode at the ratio 1.2 and lower Weber fractions (dots: *r* = -.54, *p* = .01; pictures: *r* = -.33, *p* = .15).

In a previous study, we also computed an index of the cerebral response across all numerical ratios and we observed that this index correlated with ANS acuity (Guillaume et al., 2018). To replicate this finding, we computed this index also in the current study. More concretely, we fitted a linear regression for both conditions, for each individual, predicting the Oz amplitudes at 5 Hz across the numerical ratios. We then extracted the coefficients relative to the numerical ratio (i.e., the slope) for every participant, with steeper slopes reflecting greater neural sensitivity to numerical discrimination. We excluded three individuals in total with negative regression slopes in either the dots or pictures condition. This exclusion criterium was based on a previous study by Guillaume et al. (2018; see Figure 4) as well as on the current group-level findings (see Figure 3), indicating that on average the magnitudes of the frequency-tagged medial occipital responses linearly increased with the magnitudes of the deviant numerosity. Such a linear increase between the EEG signals and numerical ratios is expected as it directly reflects behavioural performances on explicit number comparison tasks and thereby the architecture of the ANS. It thus substantiates the claim that the present EEG signals can be considered as a reliable neural index of ANS acuity. Consequently, relations between frequency-tagged cerebral responses and numerical ratios that are not positively (but negatively) increasing might reflect a failure to adequately capture numerical discrimination capacity using the current EEG paradigm. Individuals with negative regression slopes were therefore excluded from the present correlation analyses. In accordance with previous findings (Guillaume et al., 2018) and consistent with the outcomes based on the EEG amplitudes recorded for the 1.2 ratio, higher scores on the math fluency task were associated with significantly more positive linear regression slopes in both the dots (*r* = .56, *p* = .017) and the pictures (*r* = .50, *p* = .034) conditions. Moreover, lower Weber fractions were related to significantly steeper positive linear regression slopes in the dots (*r* = -.53, *p* = .024), but not the pictures (*r* = .11, *p* = .67) conditions. The linear regression slopes correlated across the two conditions (*r* = .52, *p* =.027), suggesting that neural responses were similarly sensitive to the numerical ratio across stimuli formats.
